# Molecular cloning and functional characterization of the sex-determination gene *doublesex* in the sexually dimorphic broad-horned beetle *Gnatocerus cornutus* (Coleoptera, Tenebrionidae)

**DOI:** 10.1038/srep29337

**Published:** 2016-07-11

**Authors:** Hiroki Gotoh, Mai Ishiguro, Hideto Nishikawa, Shinichi Morita, Kensuke Okada, Takahisa Miyatake, Toshinobu Yaginuma, Teruyuki Niimi

**Affiliations:** 1 Graduate School of Bioagricultural Sciences, Nagoya University, Chikusa, Nagoya 464-8601, Japan; 2 Division of Evolutionary Developmental Biology, National Institute for Basic Biology, 38, Nishigonaka, Myodaiji, Okazaki 444-8585, Japan; 3 Graduate School of Environmental and Life Science, Okayama University, Okayama 700-8530, Japan; 4 Department of Basic Biology, School of Life Science, SOKENDAI (The Graduate University for Advanced Studies), 38 Nishigonaka, Myodaiji, Okazaki 444-8585, Japan

## Abstract

Various types of weapon traits found in insect order Coleoptera are known as outstanding examples of sexually selected exaggerated characters. It is known that the sex determination gene *doublesex* (*dsx*) plays a significant role in sex-specific expression of weapon traits in various beetles belonging to the superfamily Scarabaeoidea. Although sex-specific weapon traits have evolved independently in various Coleopteran groups, developmental mechanisms of sex-specific expression have not been studied outside of the Scarabaeoidea. In order to test the hypothesis that *dsx*-dependent sex-specific expression of weapon traits is a general mechanism among the Coleoptera, we have characterized the *dsx* in the sexually dimorphic broad-horned beetle *Gnatocerus cornutus* (Tenebrionidea, Tenebirionidae). By using molecular cloning, we identified five splicing variants of *Gnatocerus cornutus dsx* (*Gcdsx*), which are predicted to code four different isoforms. We found one male-specific variant (GcDsx-M), two female-specific variants (GcDsx-FL and GcDsx-FS) and two non-sex-specific variants (correspond to a single isoform, GcDsx-C). Knockdown of all Dsx isoforms resulted in intersex phenotype both in male and female. Also, knockdown of all female-specific isoforms transformed females to intersex phenotype, while did not affect male phenotype. Our results clearly illustrate the important function of *Gcdsx* in determining sex-specific trait expression in both sexes.

## Sexually dimorphic weapons in beetles

Sexually selected weapon traits are among the most prominent morphological characteristics in animals^1^. Various types of weapon traits in Coleoptera are known as some of the outstanding examples of sexually-selected exaggerated characters^2^,^3^. Weapon characters usually express in a sex-specific (mostly male-specific) manner and function in individuals combat over limited resources^1^,^2^,^3^,^4^. Although the developmental mechanisms of the sex-specific expression of weapon traits have long remained elusive, recent progress using molecular tools in several beetle species revealed that the sex-determination gene *doublesex* plays an important role in sex-specific expression of weapon traits^3^,^5^,^6^,^7^.

### The sex-determination gene *doublesex*

*doublesex* (*dsx*) is known as a key downstream gene in the sex-determination cascade^8^. In holometabolous insects studied to date, *dsx* has at least two isoforms which are expressed in a sex-specific manner. That is, one isoform expresses only in males and functions in male differentiation, and another expresses only in females and functions in female differentiation. Dsx regulates development of both primary sexual traits (e.g. gonads and genitals) and secondary sexual traits (e.g. sex combs in male *Drosophila melanogaster*)^8^,^9^,^10^. In weaponed beetles, the function of *dsx* has been examined in four species: *Onthophagus taurus* and *O. sagitarius* (Scarabaeoidea, Scarabaeidae^5^), *Trypoxylus dichotomus* (Scarabaeoidea, Scarabaeidae^6^), and *Cyclommatus metallifer* (Scarabaeoidea, Lucanidae^7^). Although it is unlikely that the weapons of these beetles share a common evolutionary origin^11^, all have their sex-specific expression organized by Dsx (reviewed in^3^,^12^). However, all of these beetles are also closely related, belonging to the superfamily Scarabaeoidea, and functional studies of *dsx* in weapon trait expression outside of the Scarabaeoidea have been limited.

### *Gnatocerus cornutus*: sexually dimorphic weapon traits and strength as an experimental model

**Figure 1:**
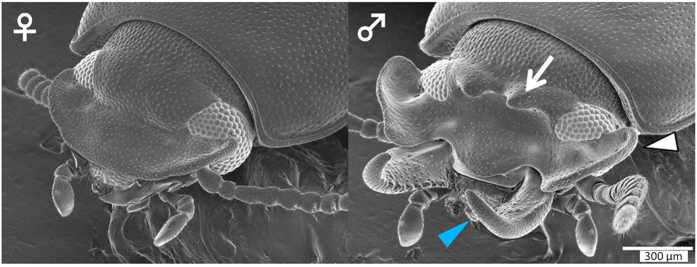
Adult female and male of *Gnatocerus cornutus.* (Left) Scanning electron microscope (SEM) image of adult female *G. cornutus*. (Right) SEM image of adult male *G. cornutus*. A pair of elongated mandibles, enlarged genae, and a pair of small head horns in the male are indicated by a light blue arrowhead, white arrowhead and white arrow respectively.

The broad-horned beetle *Gnatocerus cornutus* is a sexually dimorphic, weaponed beetle belonging to the superfamily Tenebrionidea and family Tenebrionidae. In this species, males have well-developed mandibles which are used for combat^13^. In addition to mandibles, males also possess other male-specific traits such as well-developed genae and a pair of short head horns, although their function in combat is unclear^13^. In females, mandibles are never elongated like males and other traits such as genae and horns are not expressed^13^,^14^ (Fig. 1). It is a unique characteristic of this beetle that both novel (horns) and size-modified traits (mandibles and genae) are expressed in a sexually dimorphic manner.

Compared to other previously studied weaponed scarab beetles, this species is easy to rear and breed in the laboratory. It has a shorter generation time (approximately 2 months at 25 °C incubation). Also, *G. cornutus* is phylogenetically close to the model beetle species *Tribolium castaneum*^15^, whose genome information is available^16^. Both of species belong to same family Tenebrionidae, so that the *Tribolium* genome can be used as an ideal reference genome for *G. cornutus* in potential next generation sequencing analyses. On the other hand, *Gnatocerus cornutus* is distantly related to previously studied scarab beetles with weapon characters. Estimated divergence time between Tenebrionidea and Scarabaeoidea is approximately 240 million years ago^17^. Considering these characteristics and phylogenetic position (distant from other weaponed scarab beetles and close to *T. castaneum*), *G. cornutus* can be an important new model system for investigating molecular mechanisms of the sex-specific expression of weapon traits.

Here, we identify and perform expression and functional analyses of *dsx* in order to test the hypothesis that *dsx*-dependent sex-specific expression of weapon traits is a general mechanism among different groups in the Coleoptera. Our results clearly demonstrate the significant function of *dsx* in *G. cornutus* weapon development.

## Materials and Methods

### Insects

The strain of the broad horned beetle *Gnatocerus cornutus* used in a previous study^13^ was also used in this study. We kept them in the laboratory according to Okada *et al*.^13^. Briefly, larvae were kept together in plastic containers at 25 °C and at a humidity higher than 60%. We used flour (Okura-bussann, Chiba, Japan) enriched by dried yeast (Asahi food and healthcare, Tokyo, Japan) in a 9:1 ratio as food. Larvae were transferred to 24-well plates (Becton Dickinson Labware, NJ, USA) to induce pupation.

### Identification of sex-specific genome sequences in *G. cornutus* via RAPD-PCR

The sex of *G. cornutus* is indistinguishable by external morphology during larval and prepupal periods, so we developed PCR-dependent sexing methods before the molecular analyses of *dsx*. To develop PCR-based sexing method in this species we performed RAPD-PCR, using RAPD 10mer Kits (Operon Biotechnology, Tokyo, Japan). Genomic DNA (gDNA) was extracted from whole bodies of unmated adult male and female *G. cornutus* by dissecting a single leg, washing it in water, homogenizing it in 50 μl of TES (0.1 M Tris-HCL (pH 9.0), 0.1 M EDTA, 1% SDS) and incubating it at 70 °C for 30 min. Then 7 μl of 8 M K-Acetate was added and the samples left on ice for 30 min. After centrifugation, the gDNA was ethanol precipitated. The gDNA was used in as a template in PCR performed with AmpliTaq Gold 360 Master Mix (Applied Biosystems, Foster City, CA, USA) according to manufactures’ protocol. The PCR program was: 95 °C for 9 min, and 45 cycles of [94 °C for 1 min, 35 °C for 1 min and 72 °Cfor 2 min]. PCR products were amplified using from male (but not female) samples using the A-09 primer (5′-GGGTAACGCC-3′). This male-specific PCR product was isolated by electrophoresis on a 2% agarose gel (MetaPhor Agarose, FMC BioProducts, Rockland, ME, USA) and purified using the MagExtractor PCR & Gel-Clean up kit (TOYOBO, Osaka, Japan). The purified product was subcloned using a TOPO TA Cloning Kit (Invitrogen, Carlsbad, CA, USA), and sequenced using the BigDye Terminator v3.1 Cycle Sequencing kit (Applied Biosystems). From the obtained male-specific genome sequence, we designed a primer-pair for PCR dependent sexing:

Gc-Y-05; 5′-AGTGTTGACGCAAACCTATC-3′

Gc-Y-06; 5′-AGTTCTGCAGCCATATCAGT-3′

For sexing PCR, DNA was prepared as follows: whole bodies (for larvae and prepupae) or dissected legs (for adults) were washed and homogenized in 100 μl of 50 mM NaOH and incubated at 95 °C for 15 min. Then, 100 μl of 200 mM Tris-HCl (pH 8.0) was added and the samples were centrifuged at 15,000 rpm for 10 min). The supernatant was then used directly as a template for PCR. PCR was performed with AmpliTaq Gold 360 Master Mix (Applied Biosystems), under the following cycling parameters: 95 °C for 7 min, and 35 cycles of [94 °C for 1 min, 60 °C for 30 sec and 72 °C for 30 sec]. For positive control of gDNA PCR, amplification of a section of the 28S rRNA gene sequence was used as above, except with an annealing temperature of 50 °C. Primer sequences used for PCR amplification of the 28S fragment were according to Kim *et al*.^18^ as below:

28S-F: 5′- GAC TAC CCC CTG AAT TTA AGC AT -3′

28S-R: 5′- GAC TCC TTG GTC CGT GTT TCA AG -3′.

### Molecular cloning of a *doublesex* gene fragment from *Gnatocerus cornutus*

The molecular cloning a *doublesex* gene fragment was performed via PCR with degenerate primers. The template cDNA was prepared from single male or female adults. Total RNA was extracted using TRIZOL (Invitrogen, Carlsbad, CA, USA). Then, reverse transcription was performed with SuperScript II RNase H Reverse Transcriptase (Invitrogen) using 1000 ng of total RNA. Degenerate primers were designed from the *doublesex* DM and OD2 domains conserved across insects. We also cloned the *rp49* gene from *G. cornutus* for use as an internal control in PCR. Degenerate primer sequences are listed in Table 1. PCR and subsequent subcloning were performed according to Ito *et al*.^6^ with minor modifications. Briefly, amplified PCR products were separated by electrophoresis on 1% agarose gels and purified using MagExtractor gel cleaner kit (Toyobo). Purified PCR products were subcloned using the pBlueScript KS+ vector (Stratagene, La Jolla, CA, USA) and XL1-Blue competent cells. Subcloned inserts were sequenced using an automatic DNA sequencer (DNA sequencer 3130 genetic analyser; Applied Biosystems). Database searches for identified sequence homology were performed using BlastX at the NCBI server (http://blast.ncbi.nlm.nih.gov/Blast.cgi). ClustalX was used to construct a phylogenic NJ tree of a conserved DM domain of 47 amino acids from Dsx from the following coleopteran species: *Tribolium castaneum*^19^, *Onthophagus taurus*^5^, *Trypoxylus dichotomus*^6^ and *Cyclommatus metallifer*^7^. The DNA Data Bank of Japan (DDBJ)/European Molecular Biology Laboratory (EMBL)/GenBank accession number for *Gcrp49* is LC107876.

### RACE-PCR amplification of full-length *Gcdsx* transcript variants

For amplification of full-length of *Gcdsx* we performed RACE-PCR. Using total RNA isolated as above, we synthesized cDNA for RACE-PCR using the SMART RACE kit (Clontech, Mountain View, CA, USA) according to the manufacture’s protocol. We designed four gene-specific primers (primers for initial and nested PCR for both 3′ and 5′-RACE) from the sequence of the *Gcdsx* fragment described above (Table 1). PCR was performed with Advantage2 polymeerase (Clontech, Mountain View, CA USA) using the following PCR: five cycles of [94 °C for 5 sec and 72 °C for 3 min], five cycles of [94 °C for 5 sec, 70 °C for 10 sec and 72 °C for 3 min], and 25 cycles of [94 °C for 5 sec, 68 °C for 10 sec and 72 °C for 3 min]. Using 0.5 μl of PCR product from the initial PCR reaction, nested PCR was carried out under the following parameters: 40 cycles of [94 °C for 5 sec, 70 °C for 10 sec and 72 °C for 3 min]. Amplified DNA bands were then subcloned and sequenced as described previously. The accession numbers for *Gcdsx-M, Gcdsx-FS, Gcdsx-FL, Gcdsx-C1, Gcdsx-C2* are LC105647-LC105651.

### Expression analyses of *Gcdsx* variants by PCR

In order to investigate expression pattern of each *Gcdsx* variant, we performed expression analyses via PCR. We dissected heads of last instar, early and late prepupal period, and early and late pupal period *G. cornutus* and then preserved them at −80 °C until use. Prepupal and pupal stages were judged based on external appearance and pigmentation level, respectively. Using the rest of whole body, we extracted genomice DNA and determined sex for each individual using the PCR-dependent method described before. For each sex, five heads were used for RNA extraction. Total RNA was extracted with the RNeasy Mini Kit (QIAGEN, Hilden, Germany) by the auto-RNA extractor QIAcube (QIAGEN, Hilden, Germany) according to the manufacturers’ protocol. 431.2 ng of RNA was reverse transcribed as described procedure previously. Using this synthesized cDNA as a template, PCR was performed using AmpliTaq Gold 360 Master Mix (Applied Biosystems) at 95 °C for 9 min followed by 35 cycles of [94 °C for 1 min, 45 °C for 30 sec and 72 °C for 1 min].

### Functional analyses of *Gcdsx* variants via RNAi

For investigating function of *Gcdsx*, we performed analyses via RNAi. Using primer pairs designed for each region (Table 2), partial sequences of *Gcdsx* were amplified by PCR and subcloned into TOPO vector (pCR4-TOPO) with the TOPO TA cloning Kit (Invitrogen). Then, insert regions were amplified with the universal primer pair with the T7 sequence (5′- TAATACGACTCACTATAGGGAGACCACGTCCTGCAGGTTTAAACG-3′ and 5′- TAATACGACTCACTATAGGGAGACCACCGAATTGAATTTAGCGGC-3′). Amplified PCR products were then purified as previously described. dsRNAs were synthesized using the MEGAscript T7 kit (Ambion, Austin, Tx, USA). dsRNA of the *DsRed* sequence negative control sequence was synthesized by the same method. Injection of dsRNA into larvae was performed using a microinjector (FemtoJet, Eppendolf, Hamburg, Germany) with a glass needle (Natsume-Kogaku, Nagano, Japan). The concentration of the dsRNA solution was 10 μg/μl and approximately 0.10 to 0.68 μl was injected into late instar larvae. Injected larvae were reared separately to induce pupation. Eclosed adult phenotypes were observed by binocular microscope and photographed with a VHX-900 digital microscope (Keyence, Osaka, Japan).

### Scanning electron microscopy (SEM)

Magnified images of *G. cornutus* heads were captured by SEM (VE-9800, Keyence) without any pretreatment.

## Results and Discussion

### Development of PCR-based sexing method in *G. cornutus*

**Figure 2:**
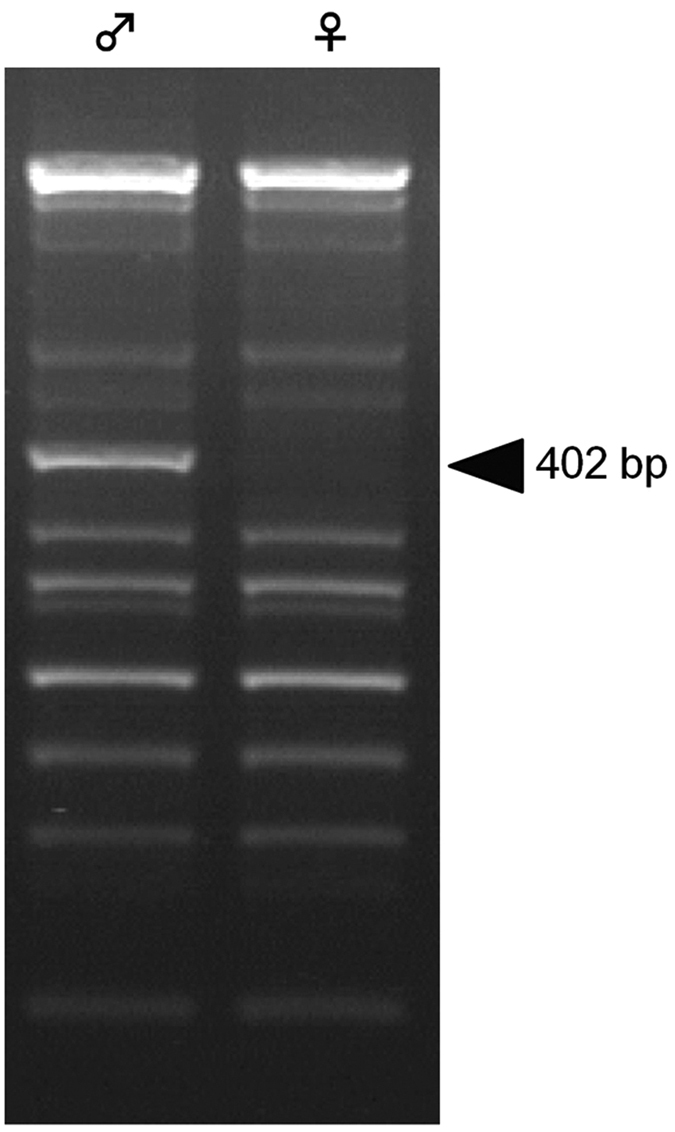
A male-specific RAPD marker of *G. cornutus.* Amplification pattern from male and female genomic DNA of *G. cornutus* using A-09 primer. Arrowhead indicates a male-specific PCR product.

The sex of *G. cornutus* is indistinguishable during the larval periods, and it is also indistinguishable in individuals that have been phenotypically disrupted by *dsx* RNAi treatment. Thus, we first developed PCR-based sexing methods. We performed PCR with twenty different 10-mer primers provided with the a RAPD kit (A-01 to A-20) using gDNA of male or female *G. cornutus*. We thus obtained a 402 bp male-specific amplicon using the A-09 primer (Fig. 2). This sequence did not show significant homology with any previously identified sequence by Blastn using the NCBI database (http://www.ncbi.nlm.nih.gov/). We then designed a pair of sexing primers (Gc-Y-05 and 06) based on this male-specific sequence, which amplified a 203 bp PCR product only when male-gDNA was used as the template (See Supplementary Fig. 1S). Considering that this species has XY sex determination system^20^, this male-specific sequence might be on Y chromosome. Thus, we used this PCR-based sexing method for identifying the original genetic sexes in phenotypically disrupted *dsx* RNAi individuals in later experiments (data not shown).

In the expression and functional analyses of sex-determination genes, it is important to know the sex of sample individuals in advance. However, some insects do not show morphological dimorphism, especially in larval and prepupal periods, which are critical periods in investigating development of sexually-selected exaggerated traits in some beetles^2^. Furthermore, when trying to identify the initial sex-determination molecular signal, it is necessary to know the sex of early embryos^21^, which are often difficult to determine morphologically. Our new male-specific primer pair enabled us to distinguish the sample sex in any developmental stage.

This PCR marker is also allows us to determine the original sex of *dsx* RNAi-treated individuals in the present study and will be used in future investigations of sex-determination mechanisms in this species.

### Identification of *dsx* in *G. cornutus*

**Figure 3:**
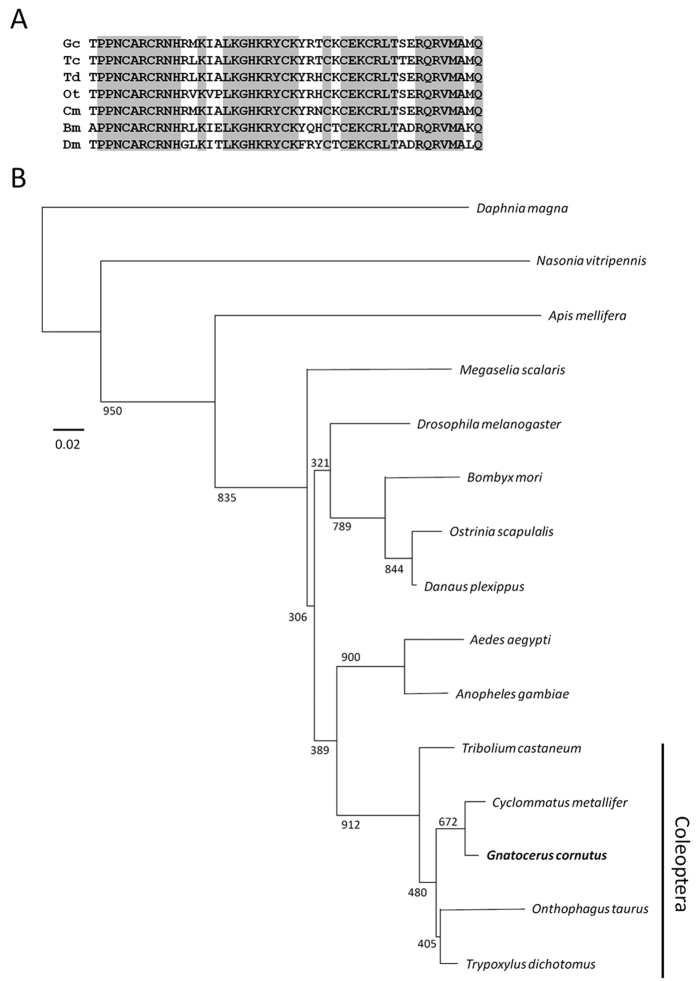
Alignment and phylogeny of GcDsx with other insect Dsx proteins. (**A**) Alignment of the conserved 47 amino acid DM domain of Dsx proteins of five coleopteran species (Gc, *Gnatocerus cornutus*; Tc, *Tribolium castaneum*; Td, *Trypoxylus dichotomus*; Ot, *Onthophagus taurus*; Cm, *Cyclommatus metallifer*) and other holometabolous insects (Bm, *Bombyx mori*; Dm, *Drosophila melanogaster*). Amino acids conserved among all seven species are highlighted in gray. (**B**) Phylogenetic tree of the 47 amino acid conserved DM domain of Dsx constructed using the neighbor-joining method with bootstrap support values (1000 iterations) indicated next to branch nodes.

First, by using degenerate PCR, we identified a partial sequence of a putative *G. cornutus dsx* homolog. We obtained the full-length clone of this gene by subsequent RACE-PCR. The putative protein coded by the identified gene sequence contains amino acids of conserved DNA binding domain (DM domain/OD1 domain) (Fig. 3A) and conserved dimerization domain (OD2 domain). The DM domain is found in all members of the Dmrt gene family, including Dsx, while the OD2 domain is specific to Dsx^22^. Phylogenetic analysis using this conserved DM domain sequence indicated that the identified sequence from *G. cornutus* was grouped with other coleopteran Dsx sequences (Fig. 3B). These results strongly suggest that the identified gene was the homolog of *dsx* from *G. cornutus*. Consequently we named this gene *Gcdsx*.

**Figure 4:**
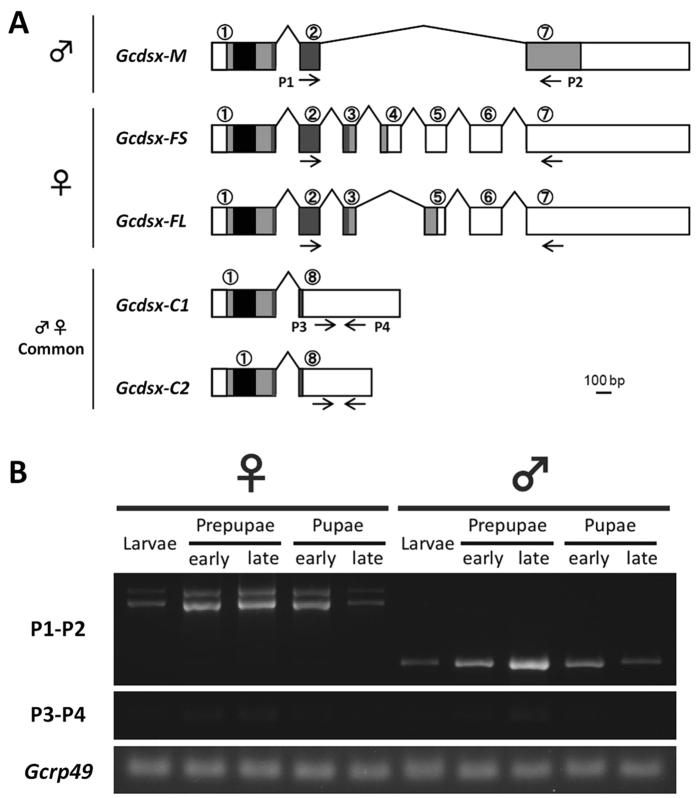
Predicted gene model and expression patterns of *Gcdsx* splicing variants. (**A**) Schematic gene model of identified *Gcdsx* splicing variants. Boxes indicate exons. White boxes indicate UTRs. Black and dark grey boxes indicate DM and OD2 domains, respectively. Light grey boxes indicate other translated portions of the proteins. Arrows indicate the primer positions used in expression analyses via RT-PCR. (**B**) Expression pattern of each splicing variant of *Gcdsx* during postembryonic development in both sexes. P1-P2 pair can amplify *Gcdsx-M*, *Gcdsx-FS* and *Gcdsx-FL*. P3-P4 pair can amplify *Gcdsx-C1* and *Gcdsx-C2*. *Gcrp49* was used as internal control.

We identified five different splicing variants of *Gcdsx* via RACE-PCR. One variant was isolated only from male, two variants were only from females and two variants were from both sexes, thus we named those variants *Gcdsx-M, Gcdsx-FS, Gcdsx-FL, Gcdsx-C1* and *Gcdsx-C2* (Fig. 4A). All of the variants have the same 5′ exon (exon1) encoding the DM domain, so all of the variants differed in the 3′ region (Fig. 4A). Exons 3, 5 and 6 were specific to *Gcdsx-FS* and *Gcdsx-FL* (Fig. 4A). *Gcdsx-FS* and *Gcdsx-FL* were distinguished by *Gcdsx-FS* specific exon 4 (Fig. 4A). We could not identify any *Gcdsx-M* specific exons. Exon 8 appeared in the non-sex-specific variants *Gcdsx-C1* and *Gcdsx-C2*, which differed in some non-coding regions, but had identical putative ORFs. The lengths of the putative ORFs (in amino acids) for the *Gcdsx* variants were: 319 (GcDsx-M), 224 (GcDsx-FS), 249 (GcDsx-FL) and 149 (GcDsx-C1 and GcDsx-C2).

Next, to examine the expression of these isoforms, we performed expression analysis between sexes by RT-PCR using the primers in listed in Fig. 4A. These results indicated that the sex-specificity of those variants were unchanged during all stages of postembryonic development (Fig. 4B). On the other hand, the expression level of sex-specific *Gcdsx* variants seemed to be greater during the prepupal period than in the late larval or late pupal periods in both sexes (Fig. 4B).

### Comparison of GcDsx isoforms with other coleopteran Dsx proteins

**Figure 5:**
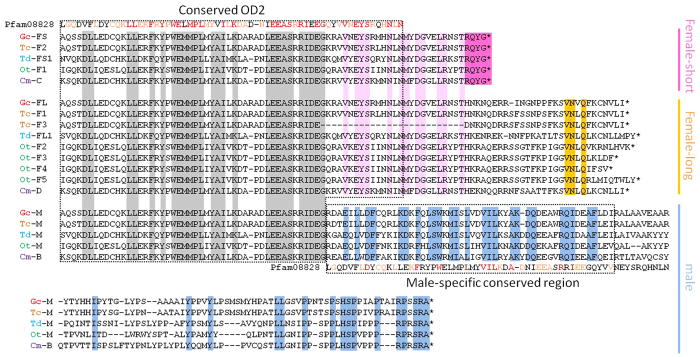
Comparison of Dsx isoforms among coleopteran species. Alignments of Dsx isoforms among five coleopteran species (Gc, *Gnatocerus cornutus*; Tc, *Tribolium castaneum*; Td, *Trypoxylus dichotomus*; Ot, *Onthophagus taurus*; Cm, *Cyclommatus metallifer*). Highlighted residues in grey, light pink, dark pink, orange and light blue indicate conserved residues among five species in OD2 region, female-specific region, female-short isoforms, female-long isoforms and male isoforms, respectively. Conserved OD2 domain (Pfam08828) sequences are aligned with Dsx sequences. Colored characters of Pfam08828 sequence indicated the residues conserved among all of five species (red) or conserved in at least two species (orange). Accession numbers of amino acid sequences of coleopteran Dsx proteins used for alignments are Gc-FS(LC105648), Tc-F2(AFQ62107), Td-FS1(BAM93344), Ot-F1(AEX92939), Cm-C(BAO23810), Gc-FL(LC105649), Tc-F1(AFQ62106), Tc-F3(AFQ62108), Td-FL1(BAM93341), Ot-F2(AEX92940), Ot-F3(AEX92941),Ot-F4(AEX92942), Ot-F5(AEX92943), Cm-D(BAO23811), Gc-M(LC105647), Tc-M(XP_001807448), Td-M1(BAM93339), OT-M(AEX92938) and Cm-B(BAO23809).

Alignment of GcDsx isoforms with other coleopteran Dsx proteins indicated that three classes of Dsx isoforms are likely to be shared within coleopteran species (Fig. 5). Considering the expression patterns of those isoforms in this species and other species^5^,^6^,^7^,^19^, the three classes can be categorized as female-specific short (Dsx-FS), female-specific long (Dsx-FL) and male-specific (Dsx-M) (Fig. 5). The Dsx-FS class can be distinguished from Dsx-FL by protein size and the presence of four highly conserved residues (RQYG) in the C-terminal end (Fig. 5), while Dsx-FL has longer C-terminal ends (Fig. 5). Conservation of amino acid sequence of Dsx-FL among species is relatively lower than the other classes, and variation in isoforms within single species can be recognized (e.g. *O. taurus* has four Dsx-FL isoforms with different C-terminal residues) (Fig. 5). Dsx-M has the longest protein sequence in all of the five coleopteran species and is easily distinguishable from Dsx-FL or Dsx-FS by lacking 14 residues in the C-terminus of the OD2 domain (Fig. 5). All Dsx-M sequences have similar protein length and share other structural characteristics, such as a well conserved RP(S/R)SRA sequence at the protein’s C-terminus, and especially possession of a conserved region just after the carboxy terminus of the OD2 domain (Fig. 5). This male-specific conserved domain shows weak similarity to the OD2 sequence. For example, the OD2 domain has two highly conserved sequences (WEMMPL) and (LEEAS(R/K)RIDEG), which are partially found in the male-specific domain. *Gcdsx-C1* and *C2* encode the same protein, GcDsx-C, which possesses a conserved DM (OD1) domain, but lack the OD2 domain. This isoform is truncated by the insertion of a stop codon at 5′ end of exon 8. *T. dichotomus* has a DsxC1 homolog with a similar size (144 aa) and characteristics (i.e. complete lack of an OD2 domain) as GcDsx-C.

In general, insect Dsx proteins have wide structural diversity on their C-terminal sides, so that it is difficult to align many regions of Dsx proteins from different insect orders. But within the coleopteran lineage, Dsx isoforms are well conserved both in structure and expression patterns^5^,^6^,^7^,^19^. Here, we propose a shared set of Dsx isoforms which may indicate common ancestry within the Coleoptera. That is, males express a single sex-specific isoform (Dsx-M) and females express two different sex-specific isoforms, one long one short (Dsx-FL and Dsx-FS, respectively) (Fig. 5).

### Functional analyses of *Gcdsx*

**Figure 6:**
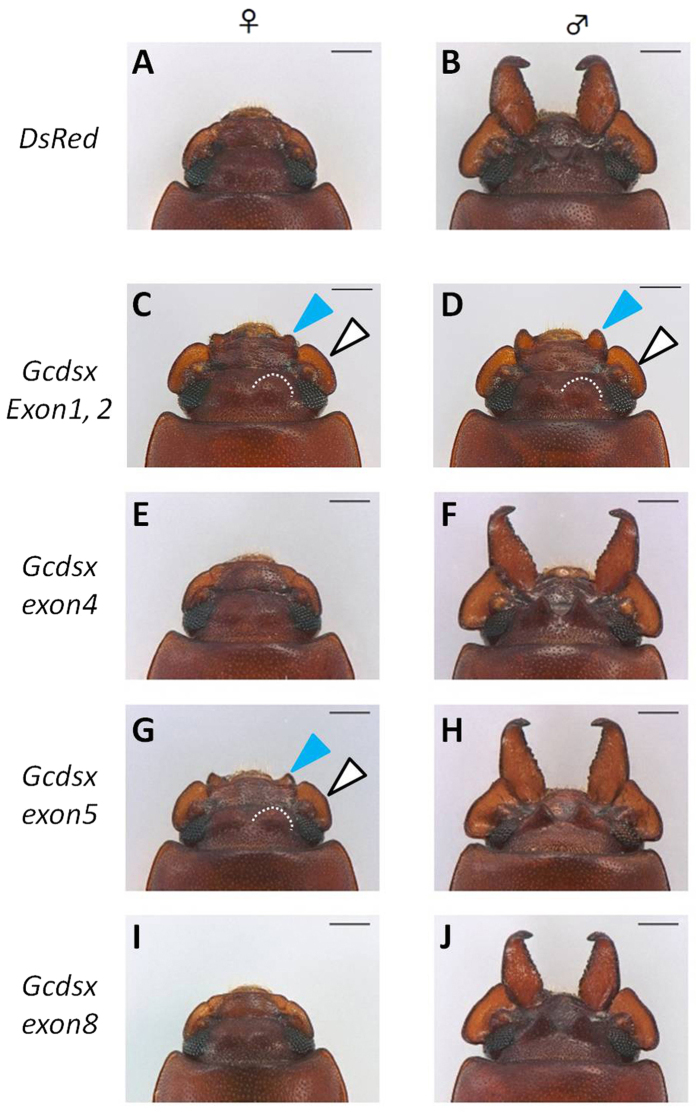
Anterior morphological phenotypes of *Gcdsx* RNAi individuals. (**A**) Negative control *DsRed* RNAi female. No male weapon traits (large mandibles, genae and a pair of small horn) are expressed. (**B**) Negative control *DsRed* RNAi male whose weapon traits (large mandibles, genae and a pair of small horns) were normally expressed the same as wild type. (**C**) *Gcdsx* exon1 and exon2 knockdown female. The mandibles became slightly larger (light blue arrowhead) and a pair of genae show significant growth (white arrowhead) compared to the control. Also, a pair of small horns was apparent between the eyes (white dashed line). (**D**) *Gcdsx* exon1 and exon2 knockdown male. Compared to control *DsRed* RNAi males, mandibles became smaller (light blue arrowhead) and the size of genae (white arrowhead) and head horns (white dashed line) was decreased. Both sexes treated with *Gcdsx* (exon1, 2) RNAi showed a similar intersexual phenotype. (**E,F**) *Gcdsx* exon4 RNAi (i.e. GcDsx-FS specific knockdown) females and males did not show any altered morphologies. (**G,H**) *Gcdsx* exon5 RNAi (i.e. both GcDsx-FS and GcDsx-FL specific knockdown) female and male. Female morphology changed to an intersexual phenotype that is characterized by slightly enlarged mandibles (light blue arrowhead), enlarged genae (white arrowhead) and a pair of horn bumps (white dashed line), while males were not affected. (**I,J**) *Gcdsx* exon8 RNAi (i.e. GcDsx-C specific knockdown) female and male. No changes in morphologies. Scale bars indicate 250 μm.

In order to reveal the function of *Gcdsx*, we performed RNAi knockdowns of *Gcdsx* isoforms. By knocking down the function of these isoforms, we clearly demonstrated their critical function in sex-specific trait development (Table 3, Fig. 6). In individuals injected with *DsRed* dsRNA as a control, none of the sexually dimorphic structures were affected in comparison with wild-type (non-injected) individuals of either sex (Fig. 6A,B). However, when we injected dsRNA against *Gcdsx* exon 1 and 2, which are shared with all of the *Gcdsx* variants, injected individuals had phenotypically disrupted morphology in both sexes. In *Gcdsx* exon1,2 RNAi females, mandibles became slightly longer and genae became wider than *DsRed* injected control individuals. Additionally a small pair of bumps was formed between the eyes, where a pair of horns normally forms in males (Fig. 6C). On the other hand, in *Gcdsx* exon1, 2 RNAi males had much smaller sexually dimorphic structures (Fig. 6D). That is, mandibles became much shorter and genae became narrower. A pair of small horns became a pair of faint bumps rather than obvious horns (Fig. 6D). In conclusion, *Gcdsx* exon1,2 knocked-down males and females showed a similar intersexual phenotype, which is likely to be a developmental default state of this species (Fig. 6C,D). These results indicate critical function of GcDsx in weapon expression in *G. cornutus* as same as in other previously studied weaponed beetles. Thus, it is suggested that *dsx* gene has been repeatedly co-opted as a developmental regulator of sexually dimorphic weapon trait formation in the various beetle lineages.

Next, we performed RNAi experiments using dsRNA against isoform-specific regions of *Gcdsx*. We designed dsRNA corresponding to exon 4 (i.e. *Gcdsx-FS* specific knockdown), exon 5 (i.e. knockdown of both female-specific *Gcdsx-FS* and *Gcdsx-FL*) and exon 8 (i.e. knockdown of both non-sex-specific *Gcdsx-C1* and *Gcdsx-C2*). Knockdown of *Gcdsx-FS* via injection of dsRNA of exon 4 and *Gcdsx-C1/C2* via injection of dsRNA of exon 8 did not affect any morphological traits in either sex (Fig. 6E,F,I,J). In contrast, knockdown of both of female-specific *Gcdsx* (*Gcdsx-FS* and *Gcdsx-FL*) via injection of dsRNA of exon 5 caused an intersexual phenotype in females (Fig. 6G), the same as with the knockdown of all *Gcdsx* isoforms (Fig. 6C), but did not affect phenotype in males (Fig. 6H). Considering that intersexual phenotypes (short mandibles, narrow genae and a pair of faint bumps) is likely to be a developmental default state, GcDsx has a critical function on sex-specific trait expression in males, and inhibition of male sex-specific traits in females. It is known that insect Dsx protein functions as transcription factor, so that GcDsx-M and GcDsx-FL appear to play a central role in the regulation of downstream genes controlling male differentiation (longer mandibles, wider genae and a pair of horn) and female differentiation (tiny mandibles, absence of genae and horns), respectively.

This and previous studies have demonstrated by isoform specific RNAi that Dsx-M and Dsx-F are essential for expression or inhibition of sex-specific weapon characters in males and females, respectively^6^,^23^. Structural differences in the C-terminal region of Dsx proteins are critical for sex-differentiation or more specifically, for sex-specific transcriptional regulation of downstream genes.

On the other hand, we could not find evidence for a function of Dsx-FS alone in sex-specific trait development, i.e. Dsx-FS isoform-specific RNAi did not affect sexually dimorphic weapon characters. There are two non-mutually exclusive interpretations of these results. First, Dsx-FS may have functions in other sexual traits such as gonad development or in different developmental stages. To date, all functional studies of *dsx* in weaponed coleopteran species have focused on postembryonic development, especially on the prepupal period when sexually dimorphic adult structures develop. Thus, although Dsx-FS type isoforms seems to be non-functional for sex-specific weapon traits via knock-down during prepupal period, it is necessary to investigate Dsx-FS function in gonad development in prepupal periods and early sex-determination including early germ cell differentiation during the embryonic stage. The second possibility is that Dsx-FL can compensate for Dsx-FS function, so that the effects of Dsx-FS knockdown were masked by the Dsx-FL isoform. In *Tribolium castaneum*, *dsxFS* RNAi affected ovarian development^19^. This result can be explained by either of those two possibilities. Further studies are necessary to reveal the functions of the two structurally different female isoforms of Dsx in coleopteran insects.

## Additional Information

**How to cite this article**: Gotoh, H. *et al*. Molecular cloning and functional characterization of the sex-determination gene *doublesex* in the sexually dimorphic broad-horned beetle *Gnatocerus cornutus* (Coleoptera, Tenebrionidae). *Sci. Rep.* **6**, 29337; doi: 10.1038/srep29337 (2016).

## Supplementary Material

Supplementary Information

## Figures and Tables

**Table 1:** Degenerate and RACE-PCR primers.

Gcdsx-degenerate-F	5′-AAYTGYGCIMGITGYMGIAAYCA-3′
Gcdsx-degenerate-R	5′-TACATIARIGGCATCATYTCCCA-3′
Gcrp49-F	5′-ACIAARMAITTYATIMGICA-3′
Gcrp49-R	5′-TGIGCIATYTCISCRCARTA-3′
5′-RACE-GSP	5′-GACTCTTCTGCAGGACATGCGGGTCTAT-3′
5′-RACE-NGSP	5′-ACTTGCAGTACCTCTTGTGGCCCTTGA-3′
3′-RACE-GSP	5′-CTCAAGGGCCACAAGAGGTACTGCAAG-3′
3′-RACE-NGSP	5′-GTCCTGCAGAAGAGTCCTTCGCCGATAC-3′

**Table 2:** Primers for dsRNA synthesis.

Gcdsx-exon1,2-F	5′-AAYTGYGCIMGITGYMGIAAYCA-3′
Gcdsx-exon1,2-R	5′-TACATIARIGGCATCATYTCCCA-3′
Gcdsx-exon4-F	5′-CCAAGAAAGGAGAATCAACGG-3′
Gcdsx-exon4-R	5′-CAACAAAGTGACGTCGCCGCTGGG-3′
Gcdsx-exon5-F	5′-TGCACAAGAACTCAACAAGAAG-3′
Gcdsx-exon5-R	5′-TTGGGACAAACGCTCCAGT-3′
Gcdsx-exon8-F	5′-TTCCAAACCGTGAATCACAA-3′
Gcdsx-exon8-R	5′-CTTGGAGCCCACTCTGAATC-3′

Gcdsx-exon1,2-F and R are identical to Gcdsx-denegenerate-F and R.

**Table 3:** Summary of *Gcdsx* RNAi experiments.

dsRNA	Sex	Injected number	Lethal stage		Adult head morphology		
			larva	pupa	female	intersex	male
exon1,2	female	18	4	1	0	13	0
	male	16	8	1	0	6	1
exon4	female	8	1	0	7	0	0
	male	28	7	0	0	0	21
exon5	female	4	1	0	0	3	0
	male	8	2	0	0	0	6
exon8	female	6	1	0	5	0	0
	male	19	11	0	0	0	8
*DsRed*	female	19	3	0	16	0	0
	male	17	1	1	0	0	15
