# Supplementary material for: Molecular cloning and functional characterization of the sex-determination gene doublesex in the sexually dimorphic broad-horned beetle Gnatocerus cornutus (Coleoptera, Tenebrionidae)
Source: Sci Rep. 2016 Jul 11;6:29337. doi: 10.1038/srep29337 (PMC4941388; doi:10.1038/srep29337)
Supplement: Supplementary Information [file srep29337-s1.pdf]

Supplementary Information

Molecular cloning and functional characterization of the sex-determination gene *doublesex* in the sexually dimorphic broad-horned beetle *Gnatocerus cornutus* (Coleoptera, Tenebrionidae)

Hiroki Gotoh[1**], Mai Ishiguro [1**], Hideto Nishikawa [1], Shinichi Morita[2], Kensuke Okada[3], Takahisa Miyatake[3], Toshinobu Yaginuma[1] and Teruyuki Niimi[1, 2, 4*]

1. *Graduate School of Bioagricultural Sciences, Nagoya University, Chikusa, Nagoya 464-8601, Japan*

2. *Division of Evolutionary Developmental Biology, National Institute for Basic Biology, 38, Nishigonaka, Myodaiji, Okazaki 444-8585, Japan*

3. *Graduate School of Environmental and Life Science, Okayama University, Okayama 700-8530, Japan*

4. *Department of Basic Biology, School of Life Science, SOKENDAI (The Graduate University for Advanced Studies), 38 Nishigonaka, Myodaiji, Okazaki 444-8585, Japan*

*Corresponding author. Division of Evolutionary Developmental Biology, National Institute for Basic Biology, 38, Nishigonaka, Myodaiji, Okazaki 444-8585, Japan. Tel.: +81-564-55-7606; fax: +81-564-55-7607.

E-mail address: niimi@nibb.ac.jp (T. Niimi).

** Contributed equally

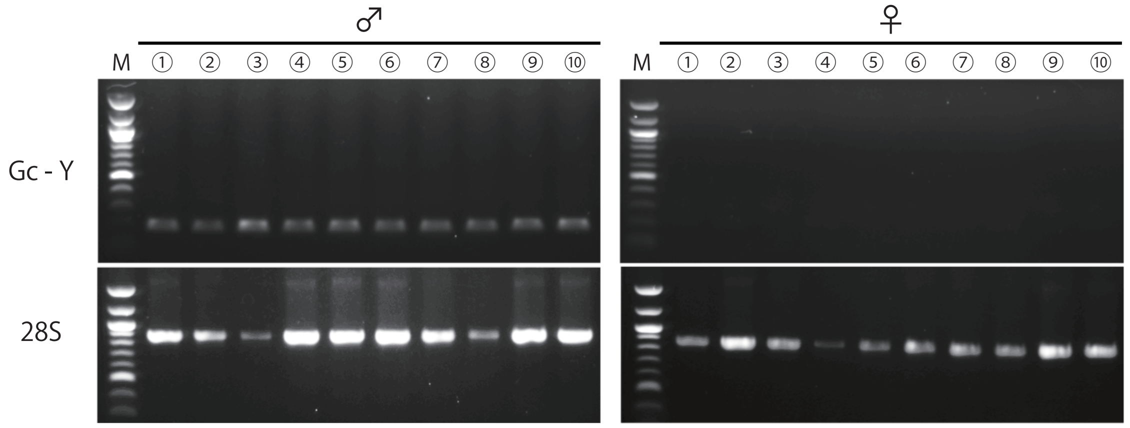

Supplementary Figure 1S. PCR-based sexing of *G. cornutus*

PCR amplification patterns of genomic DNA from ten males (left panels) and ten females (right panels) using newly developed primer pair Gc-Y. 28S rRNA gene sequence was used as positive control. M indicates 100 bp DNA ladder size marker.
